# Terahertz spectroscopy for analysis of vaterite-to-calcite crystal phase transition induced by distilled water

**DOI:** 10.1371/journal.pone.0323421

**Published:** 2025-05-12

**Authors:** Shuhei Okada, Naoya Kurahashi, Yukihiro Tanida

**Affiliations:** 1 Marketing Headquarters, Yokogawa Electric Corporation, Musashino City, Tokyo, Japan; 2 Department of Fundamental Technology, Kyoto Prefectural Technology Center for Small and Medium Enterprises, Kyoto City, Kyoto, Japan; Soongsil University, KOREA, REPUBLIC OF

## Abstract

Vaterite is a crystalline polymorph of anhydrous calcium carbonate (CaCO_3_), which exhibits relatively low stability compared to other two polymorphs, calcite and aragonite. Vaterite particles have properties such as large specific surface area, high porosity, and high solubility; hence, research has been made in wide range of applications from material additive to drug delivery. X-ray diffractometry (XRD) is capable of identifying polymorphs of calcium carbonate. However, depending on the base material and the installed state, it may not always be suitable for non-destructive observation. This study reveals that vaterite-to-calcite crystal phase transition degree can be quantified by the absorption spectrum in terahertz range. The vaterite concentration and the terahertz absorption peak intensity near 3.3 THz showed linearity with correlation coefficient of R^2^ = 0.934 in our experiment. The findings allow us to quantitatively evaluate vaterite-to-calcite crystal phase transition in non-destructive and non-invasive way.

## Introduction

Anhydrous calcium carbonate (CaCO_3_) can exist in three polymorphic forms calcite, aragonite and vaterite [1–3]. Calcite is the most stable and common polymorph which can be found in nature, particularly as a component of limestone [3–6]. It is widely used in industries as a building material, pigment, pharmaceutical, and other applications [7–9]. Aragonite is a metastable state of calcium carbonate which can be found in mollusk shells, metamorphic rocks and endoskeleton of corals [10–12]. There are few practical industrial uses of aragonite such as a pH stabilizer in aquariums and a sorptive agent to sequester pollutants such as zinc, cobalt and lead from wastewaters [13–15]. Vaterite is another metastable state of calcium carbonate which decomposes even more readily than aragonite [16–18]. Vaterite particles have properties such as large specific surface area, high porosity, and high solubility [19–21]. Past studies show that decomposition of vaterite can be induced by water and the decomposition rate is intensely related to temperature and pH [22–24]. Having these features, vaterite is considered a capable carrier for drug delivery system [25–27]. There is also a literature on the use of doped vaterite as a diagnostic additive for detecting past water intrusion in materials, providing new insights into the development of non-destructive diagnostic techniques for materials that undergo irreversible degradation by water [28]. Therefore, identifying and quantifying of vaterite in non-invasive and non-destructive way are significantly important in such applications.

The crystal polymorphs of CaCO_3_ can be clearly identified by X-ray diffractometry (XRD) [29–31], however, the surface of a target needs to be exposed for precise analysis. Furthermore, care must be taken to ensure that high X-ray flux do not cause any damage to the base material to which CaCO_3_ has been added [32–36]. Conversely, terahertz radiation, which is non-ionizing, exhibits relatively high transmittance in wide range of materials; hence, it is often considered an effective tool for non-destructive testing [37–39]. Fourier Transform Infrared Spectroscopy (FTIR) and Raman Spectroscopy are also viable tools for non-destructive analysis. A recent study also shows that combining short-wave infrared digital holography with deep learning can achieve high spatial resolution and enable real-time observation of crystal growth [40]. However, the depth of observation is often limited for incident light with a wavelength equal to or shorter than that of the long-wave infrared. It is noteworthy that terahertz also has capability of detecting crystal structure and crystallinity of materials [41–43]. Calcite shows a clear absorption peak at 3.3 THz, which is conjectured to be caused by out-of-plane vibration and in-plane vibration of the carbonate group [44]. It is also reported that aragonite shows a much lower peak at 3.3 THz compared to that of calcite, implying terahertz have capability of distinguishing various crystal shapes and structures of calcium carbonate [44,45]. However, there are no literature indicating quantitative results on vaterite measured with terahertz spectroscopy. The aim of this study is to observe difference between vaterite and calcite as well as to indicate the capability of quantifying the residual vaterite during vaterite-to-calcite crystal phase transition by terahertz spectroscopy. The test environment was designed with consideration for the application of detecting past water infiltration within materials, such as in electrical and electronic systems, with vaterite used as a diagnostic marker.

## Materials and methods

### Synthesis of vaterite

The vaterite powder used in our experiment was synthesized by aqueous CaCl_2_-Na_2_CO_3_ solution [46]. 44.0 g of calcium chloride (CaCl_2_) and 42.4 g of sodium carbonate (Na_2_CO_3_) each filled up to 400 mL by distilled water (milli-Q) were prepared, then both were mixed and stirred at speed of 200 rpm in room temperature for 30 minutes. The reaction solution was filtrated with filter pore size of 0.5 μm under reduced pressure for extraction of the sediment. The sediment was then dried under reduced pressure in temperature of 40 ºC for 12 hours and total amount of 37.0 g was collected as initial vaterite specimen in powder form. The exact same batch of specimens was used throughout our experiment to minimize the effect of material quality variation on measurements.

### Induction of crystal phase transition

The prepared vaterite powder was divided into specimens of 0.4 g each and put into glass vial filled up to 50 mL by distilled water at controlled temperature of 30 ºC. Distilled water was used to obtain fundamental data for this study. The temperature was selected within the range of standard atmospheric conditions for measurements and tests, as specified in IEC 60068-1. After each specific elapsed time of water immersion, the specimen was taken out from the vial and filtrated followed by 120 hours of drying at 30 ºC, 50%RH in atmospheric pressure.

### XRD analysis

The polymorph composition of CaCO_3_ specimens were analyzed by XRD (RINT-Ultima III, Rigaku Corp., Japan). All analysis were performed under tube voltage/current of 40 kV/ 30 mA, sampling angle step of 0.02º and scan speed of 10º/min.

### Terahertz spectrum measurement

**Fig 1:**
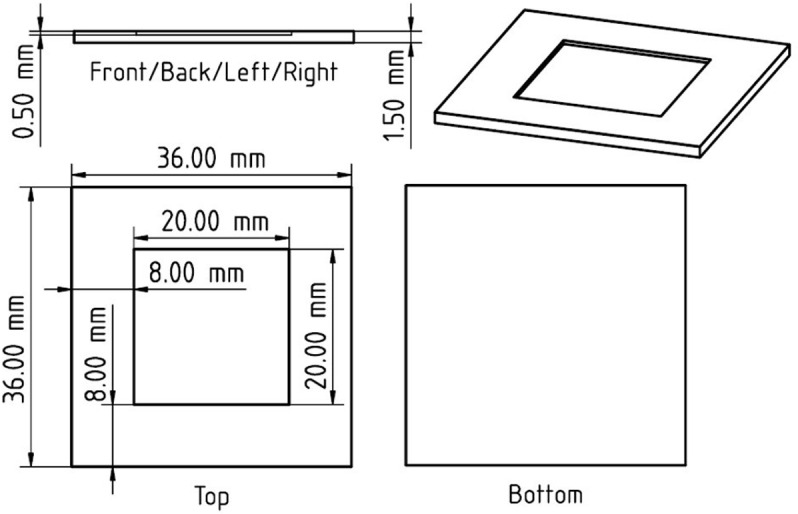
Diagram of PP plate used as a holder for terahertz transmittance measurement. Specimen is sandwiched by another PP plate with size of 36 x 36 mm and thickness of 1mm.

**Fig 2:**
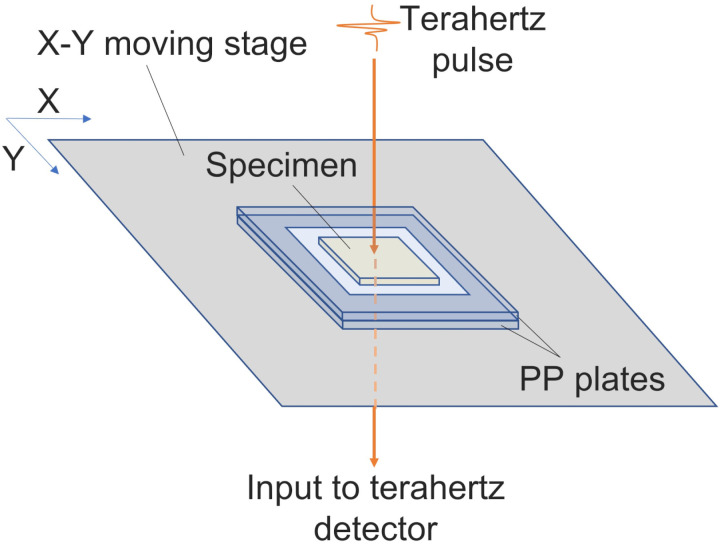
Diagram of specimen sandwiched by PP plates on a moving stage for terahertz absorption spectrum measurement.

All measurements were conducted by a terahertz time-domain spectrometer (TAS7500, Advantest Corp., Japan) in transmittance mode with a X-Y moving stage. CaCO_3_ specimen was mixed with polytetrafluoroethylene (PTFE) powder (TFW-3000FP, Sanplatec Corp., Japan) and sandwiched by two polypropylene (PP) plates (Fig 1) to conduct terahertz absorption spectrum measurements (Fig 2). The PTFE powder was used to reduce the intense absorption caused by CaCO_3_ for establishing a reasonable measurement within dynamic range of the spectrometer [47]. The pitch of the moving stage was set to 1.5 mm for both X, Y and number of 100 points at center of 15 x 15 mm area were measured to obtain an averaged spectrum. The frequency resolution and averaging number of the instrument were set to 7.6 GHz and 256 respectively. The terahertz absorption of PTFE powder itself was measured in similar process for the purpose of estimating spectra of pure CaCO_3_. The total weight and weight ratio of CaCO_3_ to PTFE powder were also measured by an electronic balance (BL-220H, Shimazu Corp., Japan) for the same purpose. The identical PP plates were used throughout the experiment. The absorption of PP was also priory measured and eliminated from each measured spectrum.

### Scanning electron microscope observation

The morphology of CaCO_3_ specimen particles were observed by scanning electron microspope (SEM) (JSM-7100F, JEOL Ltd., Japan). The specimen particles were coated with platinum by vacuum evaporation to avoid charge build-up during observation. All observations were made at acceleration voltage range of 5 k to15 kV and magnification power of 6000.

### Particle size distribution measurement

The particle sizes were measured by laser diffraction and scattering method (SALD-2300, Shimadzu Co., Japan). Ethanol was used as a dispersant and the concentration of CaCO_3_ was priorly adjusted to absorbance below 0.2 for every measurement with averaging number of 64.

## Results and discussions

### Initial state of prepared vaterite specimen

**Fig 3:**
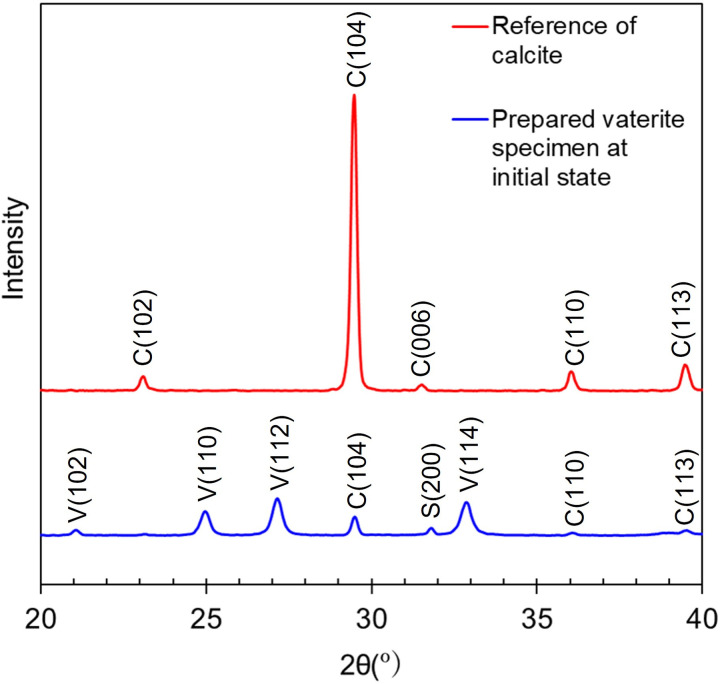
XRD pattern of prepared vaterite at initial state and commercial calcium carbonate used as reference of calcite. Crystallographic planes in parenthesis for vaterite, calcite, sodium chloride labeled in V, C, S respectively.

**Fig 4:**
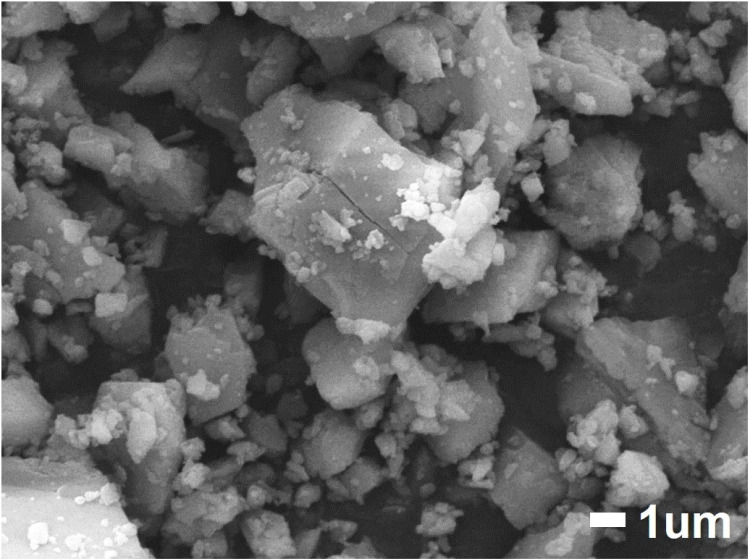
SEM image of the commercial calcium carbonate used as reference of calcite.

The XRD pattern of our prepared vaterite at initial state is shown in Fig 3 (blue line). The diffraction peaks at 2θ = 24.9°, 27.0° and 32.8° were observed which correspond to (110), (112) and (114) crystallographic planes of vaterite respectively [48,49]. The diffraction peak at 2θ = 29.4° was observed which correspond to (104) crystallographic plane of calcite [50,51]. Aragonite is known to have intense diffraction peak at 2θ = 26.3° which was not observed in our prepared vaterite [52]. The diffraction peak at 2θ = 31.8° was sodium chloride, a byproduct produced during the synthesis of vaterite [53]. The XRD pattern of commercial calcium carbonate (03000645, Hayashi Pure Chemical, Japan) was also measured showing clear peaks of calcite with no peak of other polymorphs or chemicals thus we considered it reasonable to be used as the reference of pure calcite in our measurement environment (Fig 3, red line). The SEM image is shown in Fig 4. The average diameter of particles was 6.250 μm. The purity of our initial vaterite was estimated by calculating the diffraction peak ratio of our vaterite specimen to the reference of calcite at 2θ = 29.4° which was 94.3%. The average diameter of our prepared vaterite was 4.813 μm. The reference pattern of pure vaterite was processed by subtracting the pattern of calcite from that of our prepared vaterite and multiply this by the reciprocal of this vaterite concentration which was 5.7%. These references were used to estimate residual vaterite concentrations by a pattern fitting method in the following section.

### Quantification of vaterite-to-calcite crystal phase transition

**Fig 5:**
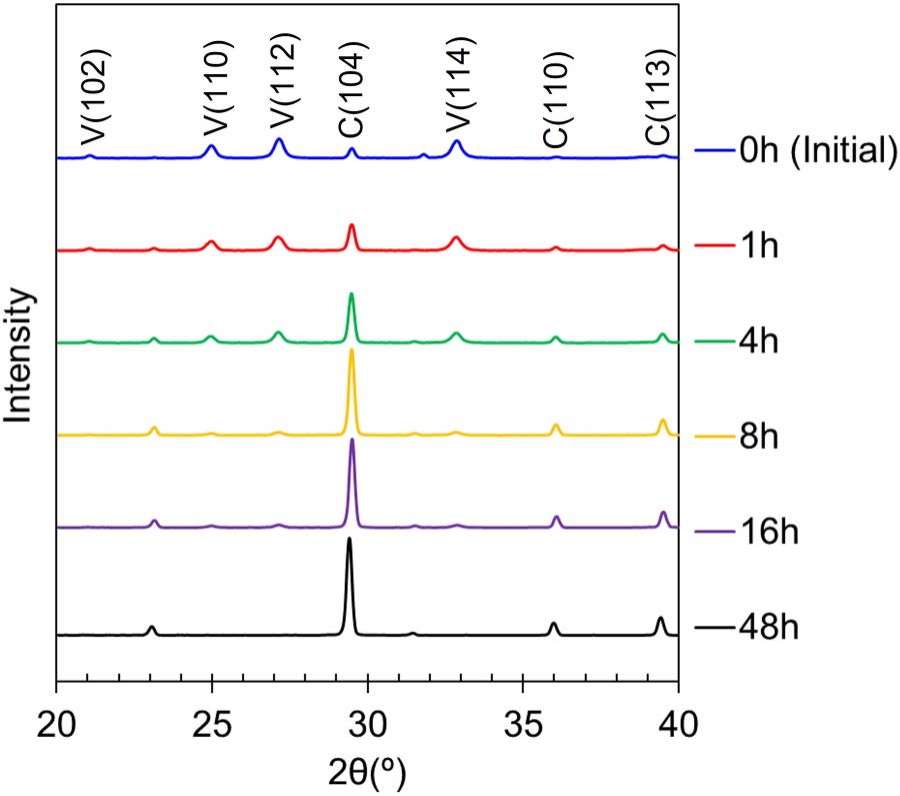
XRD pattern of prepared vaterite at initial state and after water immersion of 1, 4, 8, 16, 48 hours respectively.

Five sets of specimens were prepared for water immersion of 1, 4, 8, 16, 48 hours. Fig 5 shows the XRD patterns of our prepared vaterite at initial state and those after water immersion. The diffraction peaks of vaterite at 2θ = 24.9°, 27.0° and 32.8° decreased while diffraction peak of calcite at 2θ = 29.4° increased, indicating that vaterite-to-calcite phase transition proceeded as water immersion time elapsed [22,48–51].

Crystallographic planes in parentheses for vaterite and calcite labeled in V and C respectively.

**Fig 6:**
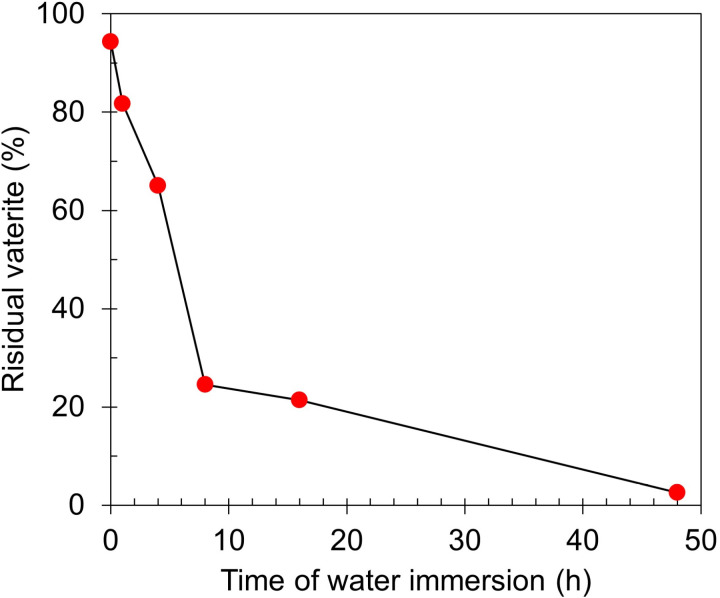
Residual vaterite concentration of vaterite specimen at different water immersion time.

Fig 6 shows the residual vaterite concentrations after water immersion. The Nelder-Mead direct search method was used for estimation of residual vaterite by fitting the scaled pure vaterite and pure calcite reference patterns to each measured pattern within range of 2θ = 20° to 40° [54,55]. The ratio of the scaling values after optimization was considered as the concentration. The intensity errors were all below 3% at (112) crystallographic plane of vaterite and (104) crystallographic plane of calcite.

### Evaluation of terahertz absorption spectrum

**Fig 7:**
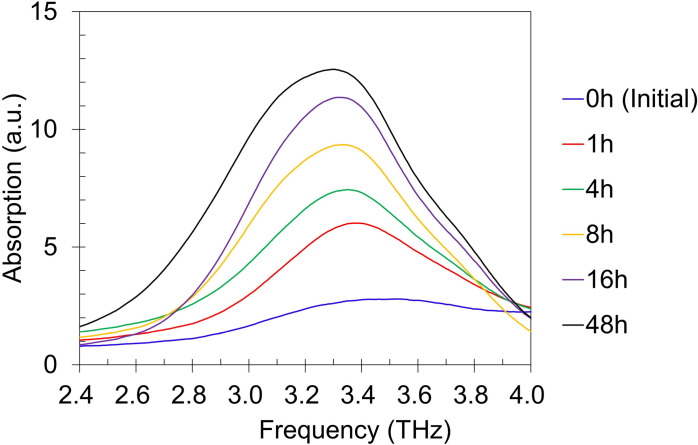
Terahertz absorption spectra of vaterite specimens before and after water immersion at 30 ºC for 1, 4, 8, 16, 48 hours.

**Fig 8:**
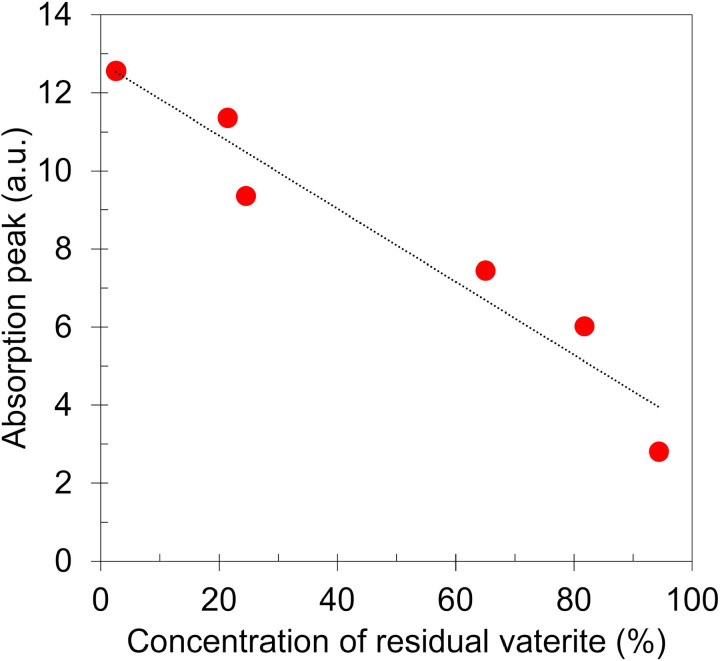
Concentration of residual vaterite vs terahertz absorption peak intensity near 3.3 THz.

Fig 7 shows terahertz absorption spectra of vaterite before and after being immersed in distilled water at 30 ºC for 1, 4, 8, 16, 48 hours. The absorption peak of calcite near 3.3 THz increased as water immersion time elapsed [22,45,46]. Fig 8 shows the correlation between concentration of residual vaterite and terahertz absorption peak near 3.3 THz for each specimen showing linearity with correlation coefficient of R^2^ = 0.934.

**Fig 9:**
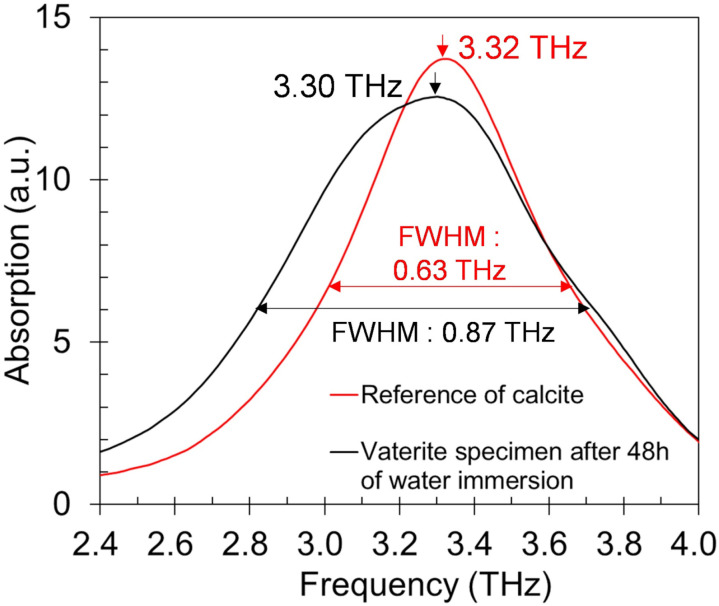
Terahertz absorption spectrum of 48-hour water immersed vaterite specimen and that of the commercial CaCO_3_ used as reference of calcite.

Although fair correlation between vaterite concentration and terahertz absorption peak intensity being observed, the spectra of water-immersed vaterite specimens showed rather broader and lower frequency peak compared to the spectrum of the commercial CaCO_3_ used as reference of calcite. As shown in Fig 6, phase transition to calcite is nearly complete after 48 hours of water immersion. However, the peak of vaterite specimen after 48 hours of water immersion and that of commercial CaCO_3_ were 3.30 THz and 3.32 THz respectively and the full width at half maximum (FWHM) of those were 0.867 THz and 0.631 THz respectively (Fig 9).

**Fig 10:**
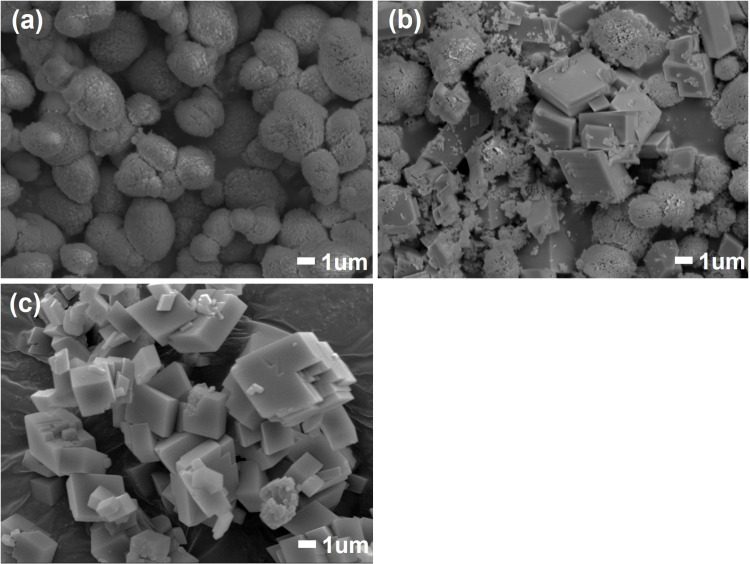
SEM images of CaCO_3_ particles. (a) The initial state of prepared vaterite specimen; (b, c) after 16 and 48 hours of water immersion.

To confirm morphology of particles, SEM observation and particle size distribution measurements were made for vaterite specimens at initial, 16 and 48 hours after water immersion. The particles transformed from spherical to cuboidal (Fig 10) and the average particle diameter grew from 4.813 μm at initial to 19.590 μm after 48 hours of water immersion (other diameter properties on Table 1).

**Table 1:** Particle size distribution of vaterite specimen.

Water immersion time of specimen (h)	Average (μm)	D25 (μm)	D50 (μm)	D75 (μm)	D90 (μm)
0 (initial)	4.813	3.303	5.272	7.625	10.455
16	15.915	9.976	17.994	29.820	46.837
48	19.590	11.554	20.437	36.232	61.342
48 (after milling)	2.663	1.827	3.163	4.758	6.506

**Fig 11:**
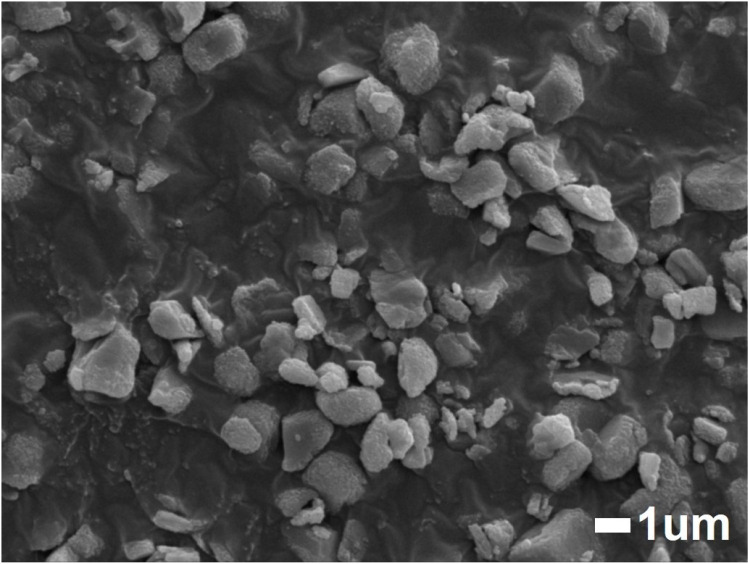
SEM image of 48-hour water immersed vaterite specimen after milling.

**Fig 12:**
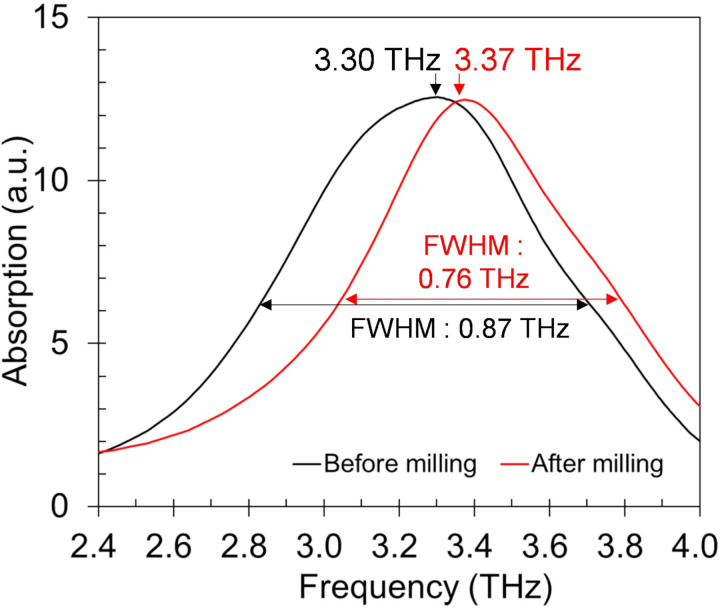
Terahertz absorption spectra of 48-hour water immersed vaterite specimen before and after milling.

In our terahertz measurement, the concentration of CaCO_3_ in PTFE powder was controlled within 4.5 to 4.9 wt % for every specimen, thus we assumed that uniformity of refractive index was impaired by large sized CaCO_3_ particles which led to intense phase incoherence of terahertz beam at the detector causing the spectra to distort [56,57]. To verify this, the specimen after 48 hours of water immersion was put to a jet mill (NJ-50, Aishin Nano Technologies, Japan) to reduce its particle sizes. After milling, the particle diameter was reduced from 19.590 μm to 2.663 μm in average (other properties on Table 1). The SEM image of the jet milled particles is on Fig 11. As a result, the broadness of terahertz absorption spectrum narrowed from FWHM of 0.87 THz to 0.76 THz, and its peak frequency shifted from 3.30 THz to 3.37 THz (Fig 12).

**Fig 13:**
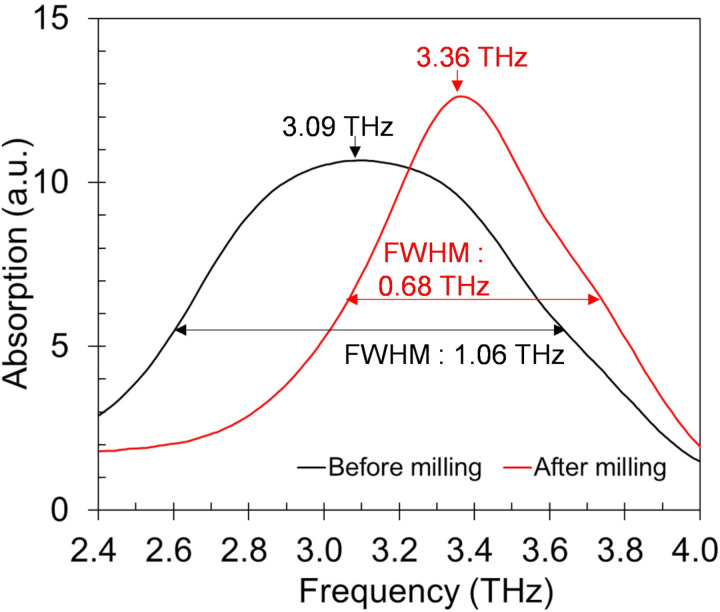
Comparison in terahertz absorption spectra of large particle sized commercial CaCO_3_ specimens before and after milling.

**Fig 14:**
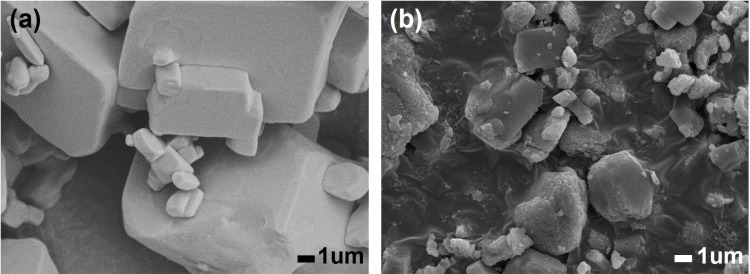
SEM images of large particle sized commercial CaCO_3_. (a) Before milling; (b) after milling.

To support the theory, we also analyzed another commercial CaCO_3_ (03000625, Hayashi Pure Chemical, Japan) having relatively large particle size with average diameter of 19.590 μm (other properties on Table 1). The peak frequency was 3.08 THz and the FWHM of spectrum was 1.06 THz showing much broader and peak shifted spectrum compared to any specimen we had observed previously, however, after milling the particles to average diameter of 2.663 μm (other properties on Table 2), the spectrum became sharp with FWHM of 0.68 THz and its peak frequency shifted to 3.36 THz (Fig 13). The SEM image of particles before and after milling is shown in Fig 14.

**Table 2:** Particle size distribution of large particle sized commercial CaCO_3_ before and after milling.

Processing	Average (μm)	D25 (μm)	D50 (μm)	D75 (μm)	D90 (μm)
none (initial)	26.823	20.920	26.679	34.054	42.697
after milling	2.685	1.791	2.936	4.374	6.029

### Mechanism of spectrum formation

**Fig 15:**
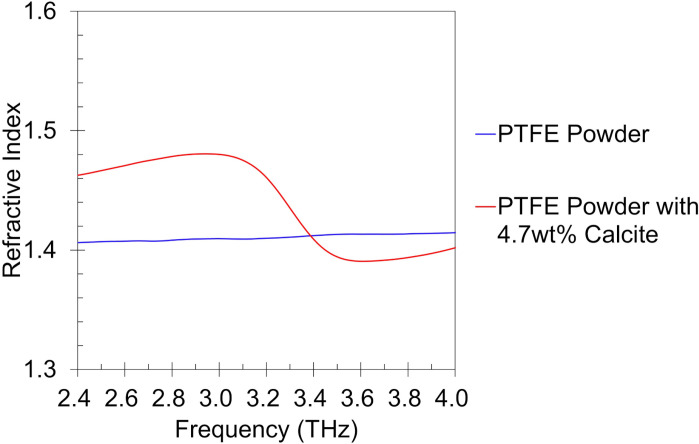
Refractive index of calcite with PTFE powder and PTFE powder alone.

Based on our experimental results, we conjectured that the broadening and peak shift of the terahertz spectra are attributed to the growth in calcite particle size and the refractive index discrepancy between calcite and the PTFE powder. However, the non-uniform geometry of the calcite particles makes precise simulation challenging. To examine the validity of this hypothesis, we measured the refractive indices of pure PTFE powder and PTFE powder containing 4.7 wt% of commercial CaCO₃ which was used as a reference of calcite (Fig 15). The averages in range of 2.4 to 4.0 THz were 1.41 and 1.44 respectively which indicate calcite has larger refractive index in average compared to the PTFE powder in this frequency range. The maximum difference between the two refractive indices was 0.071 at 2.92 THz.

**Fig 16:**
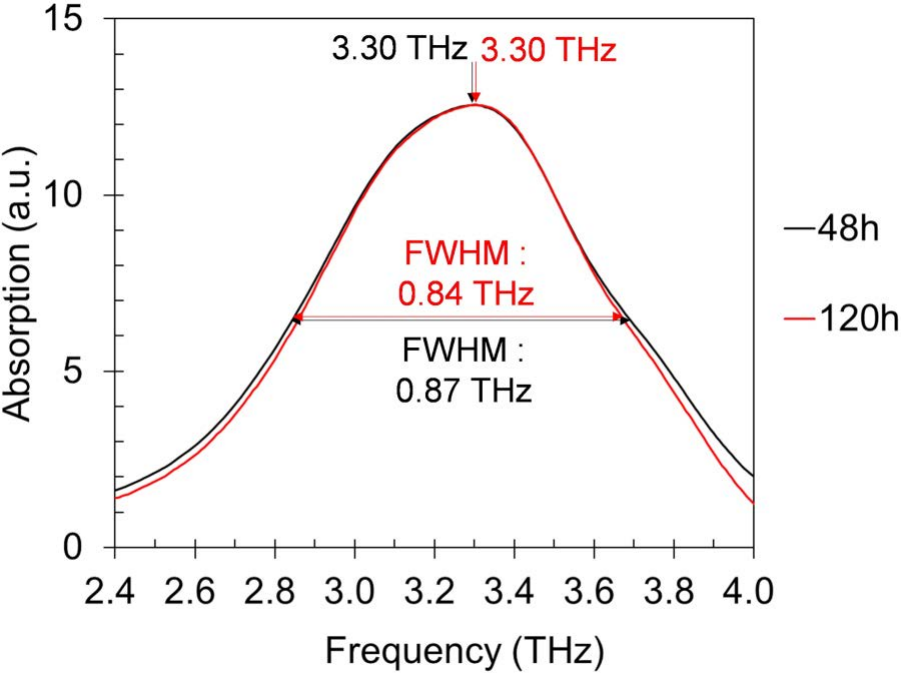
Terahertz absorption spectra of 48 and 120-hour water immersed vaterite specimen.

These overall results indicate that 2.92 THz, at which calcite exhibits the highest refractive index, is likely to cause the most severe phase interference inducing a spurious peak and/or broadening of the spectrum due to overlap with the calcite peak at 3.3 THz. The analysis suggests that as particle size increases and as particle distribution becomes more heterogeneous, spectral distortion becomes more pronounced. A previous study shows that the growth of calcite particle size occurs via a dissolution-reprecipitation mechanism, eventually reaching a saturation point [58]. Another study reports that calcite maintains a stable morphology in water [59]. Using the specimen that had been immersed in water for 48 hours, we conducted an additional experiment by extending the immersion time to a total of 120 hours. The particle size showed a slight decrease from an average diameter of 19.590 to 17.912μm (other properties on Table 3), which is conjectured to result from fragmentation of larger particles due to internal stress caused by repeated wetting and drying [60,61]. At any rate, no significant changes in the spectrum were observed compared to the period during which the crystal transition was taking place (Fig 16).

**Table 3:** Particle size distribution of vaterite specimen after immersion time of 120 hours.

average (μm)	D25 (μm)	D50 (μm)	D75 (μm)	D90 (μm)
17.912	9.650	18.737	37.082	60.541

## Conclusions

Vaterite-to-calcite crystal phase transition was observed by monitoring the terahertz absorption spectrum in the frequency range of 2–4 THz. The residual vaterite concentration and the terahertz absorption peak intensity near 3.3 THz showed a fair linearity with correlation coefficient of R^2^ = 0.934 in our experiment. However, the morphological transformation of CaCO_3_ particles caused the terahertz absorption spectra to broaden and peak shift in this frequency range. The average particle diameter of CaCO_3_ and the FWHM of terahertz absorption spectrum increased from 4.813 to 19.590 μm and 0.76 to 0.87 THz respectively after 48 hours of phase transition in distilled water at temperature of 30 ºC. We conjecture that uniformity of refractive index was impaired by large sized CaCO_3_ particles which led to incoherence of terahertz beam at the detector causing the spectra to distort. Further study with simulations on scattering and phase incoherence are necessary to evaluate the influence on the obtained terahertz absorption spectra, which was not covered in this work. Nevertheless, it is noteworthy that our findings provide a new method to quantitatively evaluate vaterite-to-calcite crystal phase transition in non-destructive and non-invasive way.
